# UHPLC-MS-MS analysis of oxylipins metabolomics components of follicular fluid in infertile individuals with diminished ovarian reserve

**DOI:** 10.1186/s12958-021-00825-x

**Published:** 2021-09-14

**Authors:** Chengcheng Liang, Xiaole Zhang, Cong Qi, Hui Hu, Qinhua Zhang, Xiuxian Zhu, Yonglun Fu

**Affiliations:** 1 Shanghai University of Traditional Chinese Medicine, Shanghai, 201203 China; 2 Department of Gynecology, Shuguang Hospital affiliated to Shanghai University of Traditional Chinese Medicine, 201203 Shanghai, China; 3 Shanghai First Maternity and Infant Hospital, Tongji University School of Medicine, Shanghai, 201204 China

**Keywords:** Diminished ovarian reserve, Follicular fluid, In vitro fertilization, Oxylipins, Metabolomics

## Abstract

**Background:**

Diminished ovarian reserve (DOR) refers to a decrease in the number and quality of oocytes in the ovary, which results in a lack of sex hormones and a decline of fertility in women. DOR can potentially progress to premature ovarian failure (POF), which has a negative impact on women's quality of life and is a major cause of female infertility. Oxidative stress is a major contributor to fertility decrease in DOR patients, affecting the follicular microenvironment, oocyte maturation, fertilization, and embryo development. Understanding intracellular signal transduction can be achieved by defining specific oxidized lipid components in follicular fluid (FF) of DOR infertile patients.

**Methods:**

The oxylipins metabolic signatures in the FF of DOR patients and females with normal ovarian reserve (NOR) enrolled for the *in vitro* fertilization (IVF) cycle were analyzed using UHPLC-MS-MS technology. Principal component analysis (PCA) and orthogonal projections to latent structure discriminant analysis (OPLS-DA) were used to analyze the derived metabolomic profiles. Pathway enrichment analysis was carried out using the Kyoto Encyclopedia of Genes and Genomes (KEGG) and MetaboAnalyst databases. Furthermore, the Spearman rank correlation coefficient was used to determine the correlation between age, FSH, AMH, AFC, oocytes retrieved, MII oocytes, fertilization, high-quality embryos, and the concentration of differential oxidized lipid metabolites in FF.

**Results:**

Fifteen oxylipins metabolites were found to be lower in the FF of DOR patients than those in the NOR group, including ±20-HDoHE, ±5-iso PGF_2α_-VI, 12S-HHTrE, 15-deoxy-Δ12,14-PGJ_2_, 1a,1b-dihomo PGE_2_, 1a,1b-dihomo PGF_2α_, 20-COOH-AA, 20-HETE, 8S,15S-DiHETE, PGA_2_, PGD_2_, PGE_1_, PGF_1α_, PGF_2α_, and PGJ_2_. The pathway enrichment analysis revealed that the 15 differentially oxidized lipid metabolites were closely related to the arachidonic acid metabolic pathway. Correlation analysis revealed that the concentration of 8 different oxidized lipid metabolites in FF was negatively correlated to FSH and positively correlated with AFC. AMH, the number of oocytes retrieved, MII oocytes and fertilization, were all positively correlated with 9 different oxidized lipid metabolites, but only one metabolite was positively correlated with the number of high-quality embryos.

**Conclusions:**

Metabolomic analysis of FF revealed that oxylipins metabolism disorders were closely related to ovarian reserve function. Among these oxylipins metabolites, arachidonic acid metabolism undergoes significant changes that may be related to oocyte development, resulting in decreased fertility in DOR patients.

**Trial registration:**

ChiCTR, ChiCTR2000038182, Registered 12 September 2020-Retrospectively registered

## Introduction

Diminished ovarian reserve (DOR) refers to a decrease in the number and quality of oocytes in the ovary, which leads to decreased female fertility and reproductive endocrine function disorders. The most common clinical manifestation of women under the age of 40 years is rare menstruation, amenorrhea, and infertility. If not treated promptly, it may progress to premature ovarian failure (POF) within 1~6 years [1]. The incidence of DOR in infertile women of different ages is estimated to range from 6% to 64% [2]. According to the US Center for Disease Control and Prevention’s national assisted reproductive technology data from 2014, DOR patients accounted for about 30% of pregnant women [3]. Due to the reduction in basic antral follicle count (AFC), the reactivity of DOR patients to ovulation-promoting drugs decreased during *in vitro* fertilization /intracytoplasmic sperm injection-embryo transfer (IVF/ICSI-ET), resulting in some phenomena such as poor ovarian responder (POR), high cycle cancellation rate, decreased number of oocytes, decreased number of high-quality embryos, and decreased cumulative pregnancy rate [4].

Oxidative stress (OS) is the phenomenon in which excessive reactive oxygen species (ROS) are produced after being stimulated *in vivo* and *in vitro*, exceeding the antioxidant capacity in the body, and there is a serious imbalance between oxidation and antioxidant system in the body, resulting in oxidative stress damage to cells and tissues [5]. ROS primarily consists of superoxide anion (O_2_^-^·), hydrogen peroxide (H2O2), hydroxyl radical (·OH), etc. Under physiological conditions *in vivo*, ROS acts as a key second messenger in intercellular signal transmission and modulates gene expression to maintain cell homeostasis*.* Excessive ROS accumulation in tissue cells, on the other hand, cause damage to proteins, nucleic acids, and lipids, resulting in the mutation or deletion of nuclear DNA and mitochondrial DNA, lipid peroxidation of cell membranes leading to changes in membrane fluidity, protein oxidative damage leading to the inactivation of important enzymes such as superoxide dismutase (SOD), catalase (CAT) and glutathione peroxidase (GSH-Px), and finally induce apoptosis and tissue structure damage [6]. The cell membrane, which is rich in polyunsaturated fatty acids, is a susceptible target of ROS attack. ROS reacts with phospholipids, enzymes, and membrane receptor-related macromolecules on the membrane surface to form malondialdehyde (MDA), 4-Hydroxynonenal, isomeric prostaglandins, and other active compounds, particularly lipid peroxides [7]. OS plays an important role in the physiological and pathological processes of the female reproductive system, which is one of the major reasons for the decline of female reproductive function. According to recent research, OS can cause ovarian endocrine dysfunction, oocyte quality decline, granulosa cell apoptosis, and then follicular atresia, which is an important reason for the decline in fertility of DOR patients, and has a significant impact on the follicular microenvironment, oocyte maturation, fertilization and embryo development [8–10].

Follicular fluid (FF) is the fluid in which oocytes exchange materials and metabolize energy with surrounding cells. Its metabolite composition can represent the level of the FF environment and, in turn, the level of oocyte metabolism. It is made up of secretions produced by peripheral granulosa cells and serum diffused by local capillaries, as well as plasma components that have crossed the blood follicular barrier, primarily hormones, growth factors, interleukins, anti-apoptosis factors, proteins, sugars, amino acids, active oxygen, and antioxidant enzymes [11, 12]. It has an impact on oocyte maturation, follicular wall rupture, fertilization, and the development of early embryos [13, 14]. Oxidative stress markers in FF are closely related to the growth, development, and maturation of oocytes. Infertility caused by DOR has long been an inescapable problem in reproductive medicine, and changes in associated metabolites in FF indicate the quality of oocytes. As a result, studying the oxylipins metabolomics in FF of infertile patients with DOR is useful.

Metabolomics, as an integral component of system biology, mimics the research ideas of genomics and proteomics, through quantitative analysis of metabolites in organisms, and determining the relative link between metabolites and physiological and pathological changes [15]. Metabolomics has recently been used to identify potential biomarkers in FF. The study of FF metabolomics is critical for evaluating and predicting the potential of *in vitro* fertilization and oocyte embryo development. Merhi et al. [16] used metabolomics to identify biomarkers of ovarian reserve function. The results showed that advanced glycation end-products (AGE) in FF were positively correlated with the level of anti-Mullerian hormone (AMH), and AGE could be used as an important biological index for predicting ovarian reserve in ART patients. Another metabolomics study found that the concentration of glucose in the FF of DOR patients decreased significantly, whereas the concentrations of lactate and progesterone increased significantly. At the same time, glucose uptake, lactic acid production, platelet-type phosphofructokinase gene expression in granulosa and cumulus cells increased significantly, while progesterone concentration decreased significantly, implying that decreased aerobic metabolism, increased anaerobic metabolism and high progesterone stimulation of DOR oocytes may be important reasons for their quality decline and early embryo dysplasia [17]. The metabolomics of oxylipins in FF of DOR patients, on the other hand, has not been documented.

In this study, UHPLC-MS-MS technology was used to detect oxylipins metabolites in FF of DOR infertile patients using target metabolomics, to identify the oxidized lipid metabolites in FF of DOR infertile patients, to screen potential biomarkers, and to investigate the possible mechanism of oocyte quality decline in DOR, which has far-reaching implications for understanding the pathogenesis of DOR.

## Materials and methods

### Sample collection and preparation

Patients with DOR (n= 20) and women with normal ovarian reserve (NOR) (n = 20, as a control group) were recruited from the Reproductive Medicine Center of Shanghai first maternity and infant hospital, Tongji university school of medicine, between May 2020 and January 2021. The study was approved by the Ethics Committees of Shuguang hospital which is affiliated with Shanghai University of traditional Chinese medicine (No.2020-833-40-01) and it was registered with the China clinical trial registration center (ChiCTR2000038182). Before the study, all subjects signed informed consent. DOR was diagnosed in patients based on the following diagnostic criteria [18]: (1) aged 20~40 years old. (2) requires one of the following three features to be met: 1) serum levels of basic follicle-stimulating hormone (FSH): 10~30 IU/L with or without FSH/ luteinizing hormone (LH) ≥ 3; 2) serum levels of AMH: 20~25 years old (AMH≤3.0 ng/ml), 26~30 years old (AMH≤2.5 ng/ml), 31~35 years old (AMH≤1.5 ng/ml), 36~40 years old (AMH≤1.0 ng/ml); 3) basic AFC on menstrual cycle (MC) 2~3: AFC≤ 5. DOR was diagnosed by meeting (1) and any two of (2). The control group included NOR patients who were infertile solely owing to tubal factors. The following were the detailed inclusion criteria: (1) aged 20~35 years old. (2) serum levels of basic FSH < 10IU/L, serum levels of basic LH<10 IU/L, with FSH/LH < 3. (3) the number of AFC: 8 < AFC < 24. (4) serum levels of AMH: 20~25 years old (3.0ng/ml<AMH≤8.4ng/ml), 26~30years old (2.5ng/ml< AMH≤8.4 ng/ml), 31~35 years old (1.5 ng/ml < AMH≤8.4ng/ml).

The study exclusion criteria were as follows: (1) endometriosis. (2) diagnosis of the polycystic ovarian syndrome. (3) presence of a functional ovarian cyst with E_2_ >100 pg/ml. (4) history of ovarian surgery such as ovarian cyst stripping and teratoma stripping. (5) receipt of hormone treatments within the preceding 3-month period. (6) any contraindications to ovarian stimulation treatment.

To avoid the influence of the ovulation induction scheme on the metabolites in FF in this study, all recruited patients, including DOR and NOR patients, adopted a micro-stimulation strategy for ovulation induction. Letrozole (Jiangsu Hengrui Medicine Co., China) was given orally for 5 days, beginning on MC 3, with a 5 mg daily dose. We began ovarian stimulation with a daily injection of gonadotropin (Gn) until the trigger day when the dominant follicular diameter of ≥ 10 mm was confirmed by transvaginal ultrasonography after MC 6. The protocol for human menopausal gonadotropin (hMG) (Zhuhai Lizhu Pharmaceutical Trading Co., China) was used as reported previously [19]. hMG was injected intramuscularly daily in alternate doses of 150 and 225 IU (150 IU of hMG given on the first day of ovarian stimulation, 225 IU on the second day, 150 IU on the third day, and so on). The hMG initiation dose was similar in the DOR and NOR groups. After 5~7 days, serum sex hormones [FSH, LH, estrogen (E_2_), and progesterone (P)] were detected, as well as a re-examination of the vaginal ultrasound. The hMG dosage was adjusted based on the level of sex hormones in the serum as well as the number and size of developing follicles. If more than one dominant follicle with a diameter≥18mm or more than three follicles with a diameter ≥16mm were present, 3000~5000IU of human chorionic gonadotropin (hCG) (Zhuhai Lizhu Pharmaceutical Trading Co., China) was used to induce oocyte maturation. We used transvaginal ultrasound-guided follicle aspiration to retrieve oocytes 36~38 h after hCG administration. Following oocyte separation, FF obtained from 3 dominant follicles was pooled in a 15 ml centrifuge tube and centrifuged at 3000 rpm for 15 min at 4°C to remove insoluble particles and cells. The supernatant was packaged in a 2ml freezing tube, and stored at -80°C in an ultra-low temperature storage box to avoid repeated freezing or thawing.

### Serum hormone measurement and antral follicle calculation

Serum sex hormones (FSH, LH, E_2,_ and P) were detected in all patients by radio-immunoassay and transvaginal ultrasonography for basic AFC on MC 2~3.

### Metabolites extraction

A 1000 μL aliquot of each sample was accurately transferred to an Eppendorf tube. Following the addition of the isotopically-labeled internal standard mixture, the samples were vortexed for 30 s and sonicated for 5 min in an ice-water bath. SPE was used to further purify the sample. The SPE cartridges were equilibrated with 1 mL of methanol and 1 mL of water. After loading a sample (supernatant obtained as mentioned above), the cartridge was washed with 1 mL of 5% MeOH/H2O (v/v). The flow-through fraction was then discarded. Finally, the samples were eluted with 1 mL of MeOH, the eluent was evaporated to dryness under a gentle stream of nitrogen, and the eluent was reconstituted in 100 μL of 30% ACN/H2O (v/v). The clear supernatant was subjected to UHPLC-MS/MS analysis, after centrifugation (15 min, 12000 rpm, 4°C).

### UHPLC-MRM-MS analysis

The UHPLC separation was performed using an EXIONLC System (Sciex), equipped with a Waters ACQUITY UPLC BEH C18 column (150 × 2.1 mm, 1.7 μm, Waters). The mobile phases A and B were 0.01% formic acid in the water, and 0.01% formic acid in acetonitrile, respectively. The temperature of the column was set to 50°C. The temperature of the auto-sampler was set to 4°C, and the injection volume was 10 μL.

For assay development, a SCIEX 6500 QTRAP+ triple quadrupole mass spectrometer (Sciex), equipped with an IonDrive Turbo V electrospray ionization (ESI) interface was used. Typical ion source parameters were as follows: Curtain Gas = 40 psi, IonSpray Voltage = -4500 V, temperature = 500°C, Ion Source Gas 1 = 30 psi, and Ion Source Gas 2 = 30 psi.

Flow injection analysis was used to analyze the MRM parameters for each of the targeted analytes by injecting the standard solutions of the individual analytes, into the API source of the mass spectrometer. In the MRM scan mode, several most sensitive transitions were used to optimize the collision energy for each Q1/Q3 pair. The Q1/Q3 pairs with the highest sensitivity and selectivity among the optimized MRM transitions per analyte were selected as a “quantifier” for quantitative monitoring. The additional transitions served as a “quantifier” to confirm the identity of the target analytes. SCIEX Analyst Work Station Software (Version 1.6.3) and Multiquant 3.03 software (Version 20.2) were used for MRM data acquisition and processing.

### Data collection, processing and statistical analysis

The data were statistically analyzed using SPSS 25.0 software. The baseline characteristics of the study population are described. The continuous variables with a normal or near-normal distribution were expressed as the means ± standard deviations (SDs) and were analyzed by Student’s t-test, otherwise, a Mann–Whitney U test was used. The frequency (composition ratio) of the classification data was statistically described and evaluated using the chi-square test. For correlation analyses, the Spearman rank correlation coefficient was used. Statistical significance was defined as *P* < 0.05.

For the metabolomics analysis, the missing values in the raw data were filled by half of the minimum value. In addition, the overall normalization method was used for data analysis. SIMCA software (V16.0.2, Sartorius Stedim Data Analytics AB, Umea, Sweden) was used to perform multivariate analysis, principal component analysis (PCA), and orthogonal projections to latent structure discriminant analysis (OPLS-DA). The original data distribution was shown using PCA. OPLS-DA was used to examine the separation between two groups and to better understand the variables responsible for classification. The variable importance in the projection (VIP) of the first principal component obtained from the OPLS-DA analysis was determined. In the univariate analysis, metabolites with a VIP > 1.0 and *P*-value < 0.05 were considered significantly different. Moreover, the quality of the OPLS-DA model was assessed using standard parameters (R^2^Y and Q^2^). The Kyoto Encyclopedia of Genes and Genomes (KEGG) (http://www.Genome.jp/kegg/) and the MetaboAnalyst 5.0 (https://www.metaboanalyst.ca/) database were used for pathway enrichment analysis.

## Results

### Clinical characteristics and ovulation outcomes

The average age, body mass index (BMI), duration of infertility, type of infertility, bAFC, AMH, bFSH, bLH, bE_2_, P, the numbers of oocytes retrieved, MII oocytes, fertilization, and high-quality embryos of the participants are presented in Table 1. There was no significant difference between DOR and NOR groups in terms of BMI, duration of infertility, type of infertility, bLH, bE2, and P levels (*P*>0.05). The average age of the DOR group was significantly older than the NOR group (*P* <0.05), bFSH levels in patients with DOR were significantly higher than in NOR patients (*P*<0.05). However, bAFC, AMH, the number of retrieved oocytes, MII oocytes, fertilization, and high-quality embryos were significantly lower in DOR patients compared to NOR patients (*P*<0.05). This indicated that the ovarian function of the DOR group was significantly low.

**Table 1:** The demographic and clinical characteristics of patients with DOR and NOR

Item	DOR group (n= 20)	NOR group (n= 20)	*P* value
Age (year)	35.350±4.017	30.350±3.514	0.001
BMI (kg/m^2^)	21.050±2.404	20.876±1.780	0.797
Duration of infertility(year)	4.350±3.014	2.900±1.586	0.211
Type of infertility, n (%)			0.342
Primary	9(45.00)	11(55.00)	
Secondary	12 (60.00)	8(40.00)	
bFSH (mIU/ml)	11.268±3.991	6.331±1.084	0.000
bLH (mIU/ml)	3.668±2.105	3.783±1.151	0.830
bE2 (pg/ml)	55.025±43.448	43.116±14.850	0.529
P (ng/ml)	0.512±0.249	0.569 ± 0.163	0.253
bAFC (n)	2.850±1.461	12.700±3.229	0.000
AMH (ng/mL)	0.709±0.344	3.900±1.189	0.000
Oocytes retrieved (n)	3.500±1.433	14.200±4.884	0.000
MII oocytes (n)	2.850±1.496	10.350±4.146	0.000
Fertilizations (n)	2.750±1.410	9.800±4.686	0.000
High-quality embryos(n)	0.750±0.786	2.650±2.777	0.012

*DOR* Diminished ovarian reserve, *NOR* Normal ovarian reserve, *BMI* Body mass index, *bFSH* basic follicle-stimulating hormone, *bLH* basic luteinizing hormone, *bE*_*2*_ basic estrogen, *P* Progesterone, *bAFC* basic antral follicle count, *AMH* Anti-Mullerian hormone

### Multivariate analysis of metabolites

**Fig. 1:**
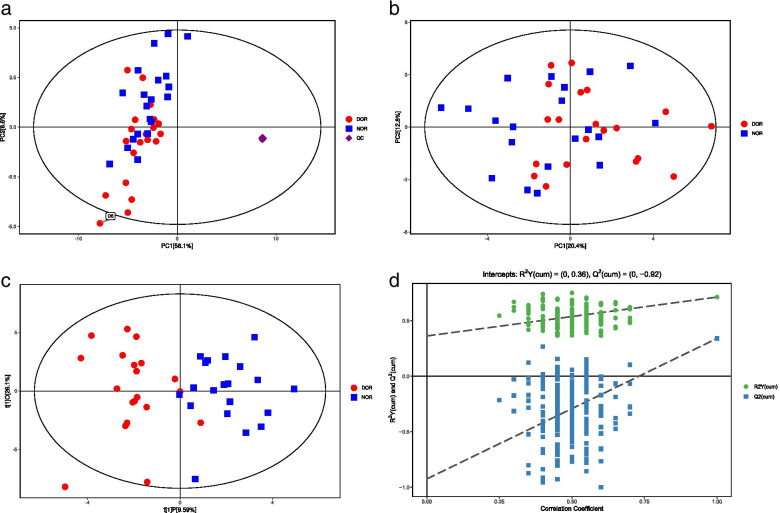
PCA score plots, OPLS-DA score plots, and corresponding validation plot of OPLS-DA results derived from FF metabolomics profiles comparing DOR and NOR. **a**. PCA score plot with QC samples. **b**. PCA score plot without the QC samples. **c**. OPLS-DA score plot. d. The OPLS-DA model’s permutation test

After relative standard deviation de-noising, 103 peaks were detected and 68 metabolites were identified in this study. The median vale was used to fill in the missing variables. The final dataset, which included information on the peak number, sample name, and normalized peak area was imported into SIMCA software for multivariate analysis. To minimize the influence of both noise and high variance of the variables, the data were scaled and logarithmically transformed. Following these changes, PCA and OPLS-DA were used to comprehensively compare the FF metabolomic profiles and determine the degree of diversity between the DOR and NOR groups. Sixteen quality control (QC) samples were included to confirm the stability and repeatability of the system, and the QCs were clustered together and separated from the study subjects' samples, indicating that the PCA was correct (Fig. 1a). The PCA score plot revealed significant differences between DOR and NOR, with one outlier located beyond the 95% Hotelling’s T-squared ellipse (Fig. 1b). As shown in Fig. 1b, the majority of DOR samples clustered to the left, while all NOR samples clustered to the right. However, one DOR sample on the right was also visible in the PCA score plots.

An OPLS-DA was performed to compare the metabolite between DOR and NOR. The OPLS-DA score plot revealed that the oxidative lipid metabolites in FF differed significantly between DOR and NOR samples (Fig.1c). As shown in Fig.1c, all NOR samples clustered to the right, while the majority of DOR clustered to the left. However, there was one DOR sample located on the right. This finding suggested that there were substantial changes in oxidative lipid metabolites between DOR and NOR patients. Furthermore, the permutation test yielded R^2^Y(cum) and Q^2^(cum) values of 0.36 and −0.8, respectively, indicating the lack of overfitting and the high predictive ability of the OPLS-DA model (Fig. 1d), and the suitability of the model for subsequent optimization analyses.

### Identification of significantly different metabolites and pathways associated with DOR

**Fig. 2:**
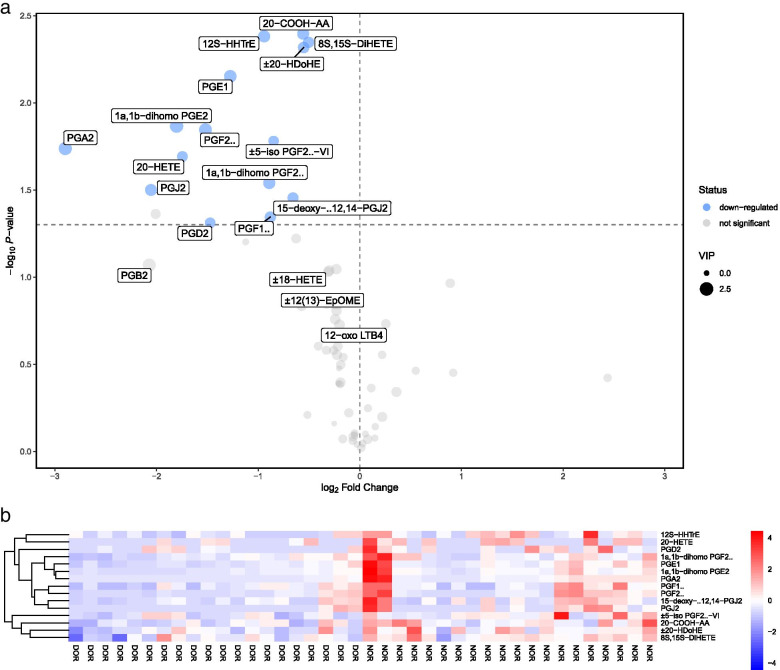
Identification of the differential metabolomics profiles of FF between DOR and NOR patients based on a volcano plot and hierarchical clustering analysis. **a**. Volcano plot, down-regulated and up-regulated metabolites in DOR compared to NOR are marked in blue and red, respectively. The X-axis represents the log2 fold change of metabolites, while the Y-axis represents the fold change of the –log10 P value determined by the Student’s t-test. The variable importance in the projection (VIP) value is represented by the dot size. **b**. Heatmap of the hierarchical clustering analysis. There are fifteen distinct metabolites presented

**Fig. 3:**
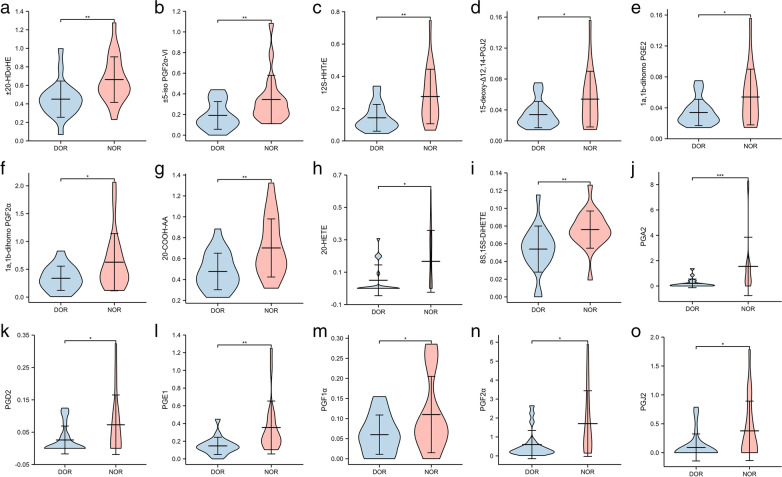
a-o The violin diagram shows the concentration of 15 different metabolite profiles in the quantitative analysis of DOR and NOR groups. The y-axis represents integration data for mass spectral concentration. Each component group is represented by the x-axis. *P <0.05, **P <0.01, ***P<0.001

In this study, 15 FF metabolites with VIP values > 1 in the OPLS-DA analysis and *P*-values < 0.05 in the univariate analysis were detected between DOR and NOR samples (Table 2), indicating that these 15 oxidized lipid metabolites were associated with ovarian reserve function. In particular, 15 metabolites were all reduced and no metabolites were increased in the DOR group compared to the NOR group. These 15 differentially oxidized lipid metabolites associated with ovarian reserve function were then summarized in a volcano plot (Fig. 2a): the 15 down-regulated metabolites (blue spots) were located on the left, while no up-regulated metabolites were found in the FF of DOR compared to the NOR group. Furthermore, a heatmap was used to distinguish the upregulated and downregulated metabolites in DOR patients compared with NOR patients (Fig. 2b). R software (Version 3.6.3) was also used to display the variations in 15 oxidized lipid metabolites in FF between the two groups. Among the differential metabolites, the levels of 15 differential metabolites were all lower in the DOR group than in the NOR control group (Fig.3).

**Table 2:** Differential metabolites in FF samples between DOR and NOR groups

Metabolites	VIP	*P*-value	Fold change	Type
±20-HDoHE	1.46468	0.00481	0.68185	Down
±5-iso PGF_2α_-VI	1.05784	0.01656	0.55571	Down
12S-HHTrE	1.76019	0.00415	0.52086	Down
15-deoxy-Δ12,14-PGJ_2_	1.27131	0.03509	0.63420	Down
1a,1b-dihomo PGE_2_	2.48940	0.01361	0.28674	Down
1a,1b-dihomo PGF_2α_	1.82901	0.02880	0.53937	Down
20-COOH-AA	1.75008	0.00401	0.67958	Down
20-HETE	1.11775	0.02031	0.29838	Down
8S,15S-DiHETE	1.38584	0.00450	0.70519	Down
PGA_2_	2.32409	0.01828	0.13424	Down
PGD_2_	1.08593	0.04879	0.36052	Down
PGE_1_	2.01038	0.00705	0.41340	Down
PGF_1α_	1.33950	0.04522	0.54329	Down
PGF_2α_	2.18401	0.01426	0.34922	Down
PGJ_2_	1.66952	0.03160	0.24083	Down

*FF* follicular fluid, *DOR* diminished ovarian reserve, *NOR* normal ovarian reserve, *VIP* variable importance in the projection

**Fig. 4:**
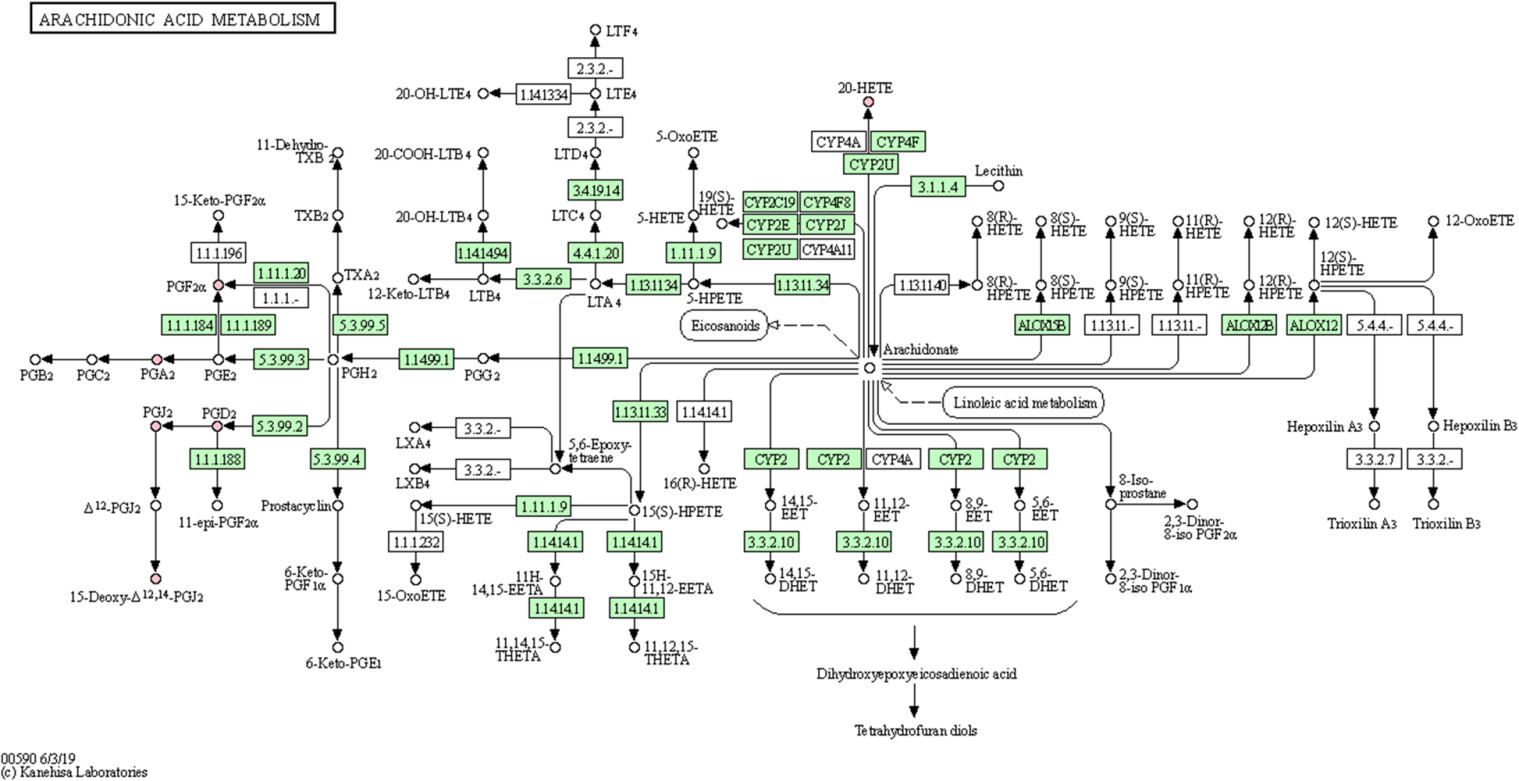
KEGG’s metabolic pathway for arachidonic acid. The pink dots represent differentially expressed metabolites involved in the arachidonic acid metabolic pathway, all of which were reduced in DOR patients. KEGG, Kyoto Encyclopedia of Genes and Genomes; DOR, diminished ovarian reserve

KEGG pathway analysis was performed to investigate the enriched metabolic pathways associated with ovarian reserve function, and an overexpression analysis of the differentially oxidized lipid metabolites was performed to reveal the biological functional background of FF metabolic disorders in infertile patients with DOR. According to the results of the KEGG Mapper Search, differentially oxidized lipid metabolites were involved in metabolic pathways, including arachidonic acid (AA) metabolism, metabolic pathways, serotonergic synapse, neuroactive ligand-receptor interaction, asthma, phospholipase D signaling pathway, vascular smooth muscle contraction, oxytocin signaling pathway, FC epsilon RI signaling pathway, African trypanosomiasis, bile secretion, and ovarian steroidogenesis. In addition, we input 15 distinct metabolites into the MetaboAnalyst 5.0 database for metabolic pathway enrichment analysis. Only one major AA metabolic pathway was discovered using the Holm *P*-value, false discovery rate (FDR), and Impact value. As a result, when combined with the findings of the KEGG database analysis, it was hypothesized that the AA metabolic pathway was closely related to DOR (Fig.4).

### Predictive value of 15 oxidized lipid metabolites in follicular fluid on ovarian reserve function

Using ROC curve analysis, the predictive value of 15 oxidized lipid metabolites in ovarian reserve function was determined, and the AUC, 95%CI, specificity, and sensitivity were obtained. At the same time, the well-known ovarian reserve function markers such as age, FSH, AMH, and AFC, were assessed and used as a reference for lipid metabolite oxidation (Table 3). The levels of ±20-HDoHE (AUC=0.782), 12S-HHTrE (AUC=0.762), 20-COOH-AA (AUC=0.758), 8S,15S-DiHETE (AUC=0.800), PGA_2_ (AUC =0.850), and PGE_1_ (AUC=0.818) in FF showed high sensitivity and specificities for the ovarian reserve function, with predictive values comparable to age (AUC=0.805). Furthermore, we found that the AUC values of FSH, AMH, and AFC were all greater than 0.95, which was superior to the conventional study to assess their clinical value [20]. The reason for the analysis could be that the patients in this study adhered to FSH, AMH, and AFC standards, demonstrating that the patients included are credible.

**Table 3:** The ROC reports between DOR and NOR group

Metabolites	AUC	95%CI	Cut-off	Sens	Spec	Sens+Spec
±20-HDoHE	0.782	0.632-0.933	0.461	0.900	0.600	1.500
±5-iso PGF_2α_-VI	0.740	0.579-0.901	0.171	0.900	0.650	1.550
12S-HHTrE	0.762	0.612-0.913	0.138	0.800	0.650	1.450
15-deoxy-Δ12,14-PGJ_2_	0.667	0.492-0.843	0.045	0.600	0.850	1.450
1a,1b-dihomo PGE_2_	0.667	0.492-0.843	0.045	0.600	0.850	1.450
1a,1b-dihomo PGF_2α_	0.680	0.512-0.848	0.372	0.650	0.650	1.300
20-COOH-AA	0.758	0.606-0.909	0.469	0.900	0.550	1.450
20-HETE	0.665	0.516-0.814	0.229	0.450	0.950	1.450
8S,15S-DiHETE	0.800	0.655-0.945	0.060	0.900	0.650	1.550
PGA_2_	0.850	0.729-0.971	0.232	0.800	0.800	1.600
PGD_2_	0.657	0.498-0.817	0.045	0.550	0.800	1.350
PGE_1_	0.818	0.687-0.948	0.170	0.800	0.700	1.500
PGF_1α_	0.644	0.470-0.818	0.057	0.750	0.500	1.250
PGF_2α_	0.735	0.576-0.894	0.660	0.650	0.800	1.450
PGJ_2_	0.675	0.535-0.815	0.127	0.500	0.850	1.350
Age	0.805	0.661-0.949	33.50	0.950	0.650	1.600
FSH	0.957	0.902-1.000	7.545	0.900	0.900	1.800
AMH	1.000	1.000-1.000	1.820	1.000	1.000	2.000
AFC	1.000	1.000-1.000	7.500	1.000	1.000	2.000

*ROC* receiver operating characteristic, *DOR* diminished ovarian reserve, *NOR* normal ovarian reserve, *AUC* area under the curve, *95%CI* 95% confidence interval, *Sens* Sensitivity, *Spec* Specificity, *Sens+Spec* sensitivity+specificity

### Correlation analysis of oxidized lipid metabolite concentrations and related parameters

Spearman correlation analysis was used to investigate the relationships between different oxidized lipid metabolites in FF and age, FSH, AMH, AFC, retrieved oocytes, MII oocytes, fertilization, high-quality embryos. The concentrations of 20-HDoHE, ±5-iso PGF_2α_-VI, 15-deoxy-Δ12,14-PGJ_2_, 1a,1b-dihomo PGE_2_, 20-COOH-AA, PGA_2_, PGD_2_, and PGE_1_ were found to be negatively correlated with FSH. Significant positive correlations were found between 20-HDoHE, ±5-iso PGF_2α_-VI, 12S-HHTrE, 1a,1b-dihomo PGE_2_, 20-COOH-AA, 8S,15S-DiHETE, PGA_2_, PGE_1_, PGF_2α_, and AMH. The concentrations of 20-HDoHE, ±5-iso PGF_2α_-VI, 1a,1b-dihomo PGE_2_, 20-COOH-AA, 8S,15S-DiHETE, PGA_2_, PGE_1_, and PGF_2α_ were positively correlated with AFC. The oocytes retrieval was positively correlated with 20-HDoHE, ±5-iso PGF2α-VI, 12S-HHTrE, 1a,1b-dihomo PGE_2_, 20-COOH-AA, 8S,15SDiHETE, PGA_2_, PGE_1_, and PGF_2α_. MII oocytes and fertilization were positively correlated with the concentrations of 20-HDoHE,12S-HHTrE,1a,1b-dihomo PGE_2_, 20-COOH-AA, 8S,15S-DiHETE, PGA_2_, PGD_2_, and PGF_2α_. Only 20-COOH-AA had a positive correlation with high-quality embryos (Table 4).

**Table 4:** Correlation analysis between the concentration of oxidized lipid metabolites and related parameters

Metabolites(ng/ml)	R/*P*	AGE	FSH	AMH	AFC	OR	MII	FER	HQE
20-HDoHE	R	-0.120	-0.555**	0.426**	0.581**	0.446**	0.457**	0.400*	0.250
	*P*	0.461	0.000	0.006	0.000	0.004	0.003	0.010	0.120
±5-iso PGF2α-VI	R	-0.175	-0.455**	0.361*	0.508**	0.361*	0.341	0.273	0.103
	*P*	0.286	0.004	0.024	0.001	0.024	0.052	0.093	0.531
12S-HHTrE	R	-0.101	-0.303	0.365*	0.252	0.347*	0.385*	0.361*	0.002
	*P*	0.535	0.057	0.021	0.117	0.028	0.014	0.022	0.989
15-deoxy-Δ12,14-PGJ_2_	R	0.024	-0.346*	0.264	0.298	0.144	0.147	0.096	-0.266
	*P*	0.882	0.029	0.099	0.062	0.376	0.365	0.556	0.097
1a,1b-dihomo PGE2	R	-0.147	-0.347*	0.547**	0.425**	0.463**	0.495**	0.497**	0.151
	*P*	0.367	0.028	0.000	0.006	0.003	0.001	0.001	0.351
1a,1b-dihomo PGF2α	R	0.052	-0.148	0.259	0.296	0.223	0.276	0.287	0.056
	*P*	0.749	0.361	0.107	0.063	0.166	0.084	0.073	0.730
20-COOH-AA	R	-0.022	-0.353*	0.390*	0.362*	0.393*	0.414**	0.425**	0.339*
	*P*	0.892	0.025	0.013	0.022	0.012	0.008	0.006	0.032
20-HETE	R	0.191	-0.386	0.327	0.447	0.510	0.436	0.412	-0.220
	*P*	0.494	0.156	0.234	0.095	0.052	0.104	0.128	0.431
8S,15S-DiHETE	R	-0.023	-0.241	0.509**	0.564**	0.452**	0.377*	0.356*	0.099
	*P*	0.889	0.145	0.001	0.000	0.004	0.020	0.028	0.553
PGA2	R	-0.149	-0.504**	0.511**	0.503**	0.408*	0.424*	0.373*	-0.192
	*P*	0.408	0.003	0.002	0.003	0.019	0.014	0.033	0.284
PGD2	R	-0.187	-0.551*	0.009	0.385	0.374	0.482*	0.463*	-0.168
	*P*	0.442	0.015	0.972	0.104	0.115	0.037	0.046	0.492
PGE1	R	-0.178	-0.481**	0.559**	0.512**	0.442**	0.437**	0.405*	-0.058
	*P*	0.278	0.002	0.000	0.001	0.005	0.005	0.011	0.726
PGF1α	R	-0.085	-0.323	0.278	0.297	0.128	0.131	0.148	-0.287
	*P*	0.644	0.071	0.124	0.099	0.485	0.475	0.418	0.111
PGF2α	R	-0.162	-0.288	0.383*	0.357*	0.344*	0.414**	0.404*	-0.074
	*P*	0.319	0.072	0.015	0.024	0.030	0.008	0.010	0.649
PGJ_2_	R	-0.100	-0.330	0.071	0.339	0.014	0.196	0.025	-0.422
	*P*	0.745	0.271	0.817	0.257	0.964	0.520	0.936	0.151

*P*, *P*-value,**P<0.01,*P<0.05; AGE, age; FSH, follicle stimulating hormone; AMH, anti-Mullerian hormone; AFC, antral follicle count; OR, number of oocytes retrieved; MII, number of MII oocytes; FER, number of fertilizations, HQE, number of high-quality embryos

## Discussion

Oxylipins, also known as lipid mediators, are a class of oxidative metabolites produced by the autooxidation of polyunsaturated fatty acids (AA, linoleic acid, alpha-linolenic acid, DHA, EPA, etc.) or by cyclooxygenase (COX), lipoxygenase (LOX), and cytochrome P450 (CYP) enzymes. Oxylipins are signal transduction molecules that participate in nearly all physiological functions of the body and play a critical regulatory role in the organism’s life activities, including inflammatory response, immune defense, endocrine regulation, oxidative stress, etc [21]. Besides, it is closely related to the occurrence and development of a variety of diseases, including tumor [22], cardiovascular disease [23], diabetes [24], lung disease [25], and Alzheimer's disease [26]. However, no studies have been conducted to investigate the relationship between oxylipins and the occurrence and development of DOR. Therefore, we performed a UHPLC-MS-MS analysis of the composition of FF oxylipins metabolites in patients with DOR and NOR. Our findings demonstrated changes in oxylipins metabolites in FF of infertile patients with DOR, as well as changes in ovarian reserve function in infertile patients with DOR. We identified 15 oxylipins metabolites that were associated with DOR, including ±20-HDoHE, ±5-iso PGF_2α_-VI, 12S-HHTrE, 15-deoxy-Δ12,14-PGJ_2_, 1a,1b-dihomo PGE_2_, 1a,1b-dihomo PGF_2α_, 20-COOH-AA, 20-HETE, 8S,15S-DiHETE, PGA_2_, PGD_2_, PGE_1_, PGF_1α_, PGF_2α,_ and PGJ_2_. The levels of the 15 differentially oxidized lipid metabolites in the FF of DOR patients were all lower than in the NOR group, and the differences were statistically significant. Based on the KEGG and Metaboanalyst database, the results of pathway enrichment analysis showed that the differential oxylipins metabolites were closely related to the AA metabolic pathway. These distinct oxylipins metabolites and the AA metabolic pathway may play a non-negligible role in DOR infertility. Dysfunctions in oxylipins metabolism in FF of DOR patients related to follicular development may provide potential diagnostic and therapeutic targets for promoting oocyte maturation.

Polyunsaturated fatty acids (PUFAs) are long-chain fatty acids with two or more double bonds, the n-3 and n-6 series fatty acids having biological significance. Imbalanced dietary intake of n-6 to n-3 PUFAs has been associated with cardiovascular and cerebrovascular diseases, cancer, inflammation, and autoimmune diseases [27]. Wathes et al. [28] demonstrated that dietary n-6 and n-3PUFAs can alter reproductive processes by acting as precursors for prostaglandin synthesis and by regulating the expression patterns of many key enzymes involved in prostaglandin and steroid metabolism. AA is an n-6 PUFA essential fatty acid that is cleaved from phospholipids by phospholipase A2 (PLA2), phosphatidyl inositol specific phospholipase C, and the fatty acylglycerol lipase. AA is then metabolized to eicosanoids via COX, LOX, or P450-dependent cyclooxygenase pathways [29]. A previous study demonstrated that high concentrations of AA derivatives in human FF at the time of oocyte retrieval significantly decreased oocyte’s ability to form pronuclei after ICSI [30]. As cell signaling intermediates, AA and its derivatives regulate cAMP activation, Ca^2+^ influx, Ca^2+^/CaM, PKC, MAPK, and PI3K/Akt, resulting in regulation of cellar growth, proliferation, and differentiation [31, 32]. Another study reported that AA is one of the most abundant polyunsaturated fatty acids [33]. The concentration of AA in FF in bovine ovaries accounts for about 2.5% of total fatty acids, whereas the concentration in plasma is 1.2% [34]. It has been reported that follicular AA regulates cumulus granulosa cells in human [35] and non-human mammals [33]. Zhang et al. [36] demonstrated that AA regulates the survival, gene expression, lipid formation, steroid production, and intracellular signaling pathways in bovine granulosa cells. This supports the hypothesis that AA in FF can directly influence the function of granulosa cells, thereby modulating follicular development and ovulation. Khajeh et al. [37] investigated the biological effects of AA in human cumulus granulosa cells (CGCs) after exposure to acetylsalicylic acid (ASA). They found that ASA treatment reduced E_2_ production, Cyp19a1 expression, GSH-Px activity, and estradiol receptor expression in CGCs. Importantly, the addition of AA abolished the ASA-induced reduction in E_2_ levels and expression of Cyp19a1, increased the antioxidant capacity of CGCs exposed to ASA by increasing GSH-Px activity, synthesis, and secretion of PGE_2_, and increasing the expression of the estrogen receptors. Li et al. [38] found that the levels of AA and its metabolites were elevated in FF of patients with PCOS, and insulin increased the generation of AA metabolites by COX-2. This is likely to be a novel molecular pathophysiological mechanism of PCOS. In the present study, we found that the level of AA metabolites in FF of patients with DOR was lower compared to its level in the NOR control group, and these decreased AA metabolites were generated via COX-2 and CYP pathway, but not LOX pathway. CYP enzymes mediate AA transformation into 20-hydroxyeicosatetraenoic acid (20-HETE) [39]. A study revealed a higher serum concentration of 20-HETE in obese women as compared to that in women with normal weight [40]. Elsewhere, 20-HETE concentration in urine and plasma of patients with metabolic syndrome was found to be higher as compared to that of control [41]. There is more evidence of a significant correlation between the level of 20-HETE in blood with BMI, whereby higher BMI denote higher levels of 20-HETE [42]. 20-HETE significantly impacts obesity associated with a high-fat diet, impaired insulin signaling, and insulin resistance (IR) [43]. Li et al. [38] found higher levels of 20-HETE in the FF of non-obese PCOS patients that underwent IVF as compared to that in the non-PCOS group, but the difference was not significant [38]. We, herein, reported lower levels of 20-HETE in FF of DOR patients as compared to that of subjects in the NOR control group. However, the correlation and mechanism between 20-HETE and DOR remain elusive.

AA is a well-known precursor of prostaglandins (PGs), which are synthesized PGs by prostaglandin-endoperoxide synthase S1 (PTGS1) or PTGS-2 previously identified as cyclooxygenase enzymes COX1 and COX2 [44, 45]. PTGS-2(COX2) is the critical prostaglandin-endoperoxide synthase in the ovary. Mounting evidence indicates that PTGS-2(COX2) deficient female mice exhibit multiple failures in female reproductive processes, including ovulation, fertilization, implantation, and decidualization [46, 47]. Prostaglandins, especially PGE_2_ and PGF_2α_, have previously been shown to regulate oocyte maturation, ovulation, and cumulus expansion [48, 49]. Additional evidence indicates that PGE_2_ plays a crucial role in protecting oocytes against oxidative stress, therefore, holds promise as a novel autocrine/paracrine player in the mechanisms that potentially drive successful oocyte maturation and oocyte survival in cow [50]. Moreover, PGF_2α_ is synthesized in response to LH surge, however, its underlying role in the ovulatory process remains to be determined [51]. Some researchers suggest that both PGF_2α_ and PGE_2_ are important in the ovulatory process [52, 53]. Pereira de Moraes et al. [54], however, concluded that PGF_2α_ alone is unable to induce ovulation in cattle. Sharma et al. [55] investigated PGE_2_ treated follicles and revealed a reduction in the attributes of apoptosis in granulosa cells, whereas PGF_2α_ saw an increase in apoptotic characteristics. Furthermore, Kemiläinen et al. [56] found a broad expression of HSD17B12 enzyme in both human and mouse ovaries, demonstrating a role for HSD17B12 in the synthesis of PGD_2_, PGE_2_, PGF_2α_, and TXB2, which are well-known prostaglandins for their regulatory role in ovarian function. Another product of the AA cyclooxygenase pathway is 15-deoxy-Δ12,14-PGJ_2_, a specific endogenous ligand for a peroxisome proliferator-activated receptor γ (PPARγ) *in vivo* [57]. PPARγ was detected in primary and secondary follicles, but was expressed highly in the large follicles, and is thought to play a role in regulating the expression of genes involved with growth, development, and/or differentiation of the follicle. Treatment of granulosa cells with 15-deoxy-Δ12,14-PGJ_2_ stimulated basal progesterone secretion but elicited no significant effect on FSH-stimulated steroid production, suggesting a potential role for PPARγ in regulating genes related to follicular differentiation [58]. Also, the PPARγ decline in response to LH is important for ovulation and/or luteinization [58]. Significantly higher levels of AA metabolites (such as PGE_2_, PGD_2_, PGJ_2_, 15-deoxy-Δ12,14-PGJ_2,_ and PGF_2α_) produced via the COX pathway were found in FF of PCOS patients as compared to those of the non-PCOS group [38]. In the present study, the levels of AA metabolites (such as PGE_2_, PGD_2_, PGJ_2_, 15-deoxy-Δ12,14-PGJ_2,_ and PGF_2α_) produced via the COX pathway in FF of DOR patients were lower than those in the NOR control group. However, the specific mechanism of DOR and the level of metabolites produced by the arachidonic acid COX enzyme metabolic pathway remain elusive.

Our analysis revealed15 types of differentially oxidized lipid metabolites in FF, possessing certain predictive value for ovarian reserve function. In particular, the levels of ±20-HDoHE, 12S-HHTrE, 20-COOH-AA, 8S,15S-DiHETE, PGA_2_, and PGE_1_ showed high sensitivities and specificities. Through Spearman correlation analysis, we also explored the correlations between the concentration of differentially oxidized lipid metabolites in FF and age, FSH, AMH, AFC, oocytes retrieved, MII oocytes, fertilization, high-quality embryos. Results demonstrated a negative correlation between the concentrations of eight lipid oxidative metabolites in FF with FSH but a positive correlation with AFC. Nine types of oxidized lipid metabolites were positively correlated with AMH, the number of oocytes retrieved, MII oocytes, and fertilization. Notably, one metabolite was positively correlated with the number of high-quality embryos. The changes in the content of oxidized lipid metabolites in FF of DOR patients suggest their potential role in oocyte development, maturation, fertilization, and early embryo development. However, it remains elusive as to how the above lipid-oxidizing metabolites play a role in FF and which metabolic regulation channels influence ovarian reserve function and oocyte quality, which warrants further exploration.

Intriguingly, our analysis showed that the differential oxidized lipid metabolites in FF of DOR patients were lower than those of NOR patients, which contradicts our original hypothesis of the search for markers of oxidized lipid metabolism in FF of DOR patients. The possible reasons may be that: First, our reproductive center routinely employed a double-cavity needle to wash oocytes, allowing DOR patients to obtain maximum levels of oocytes, which is not the case for NOR patients. As such, we insinuate the potential influence of needle washing liquid on oxidized lipid metabolites in FF. Second, the structure of some oxidized lipid metabolites in FF may be unstable; however, we made our best attempt to ensure that the processes of collecting, transporting, centrifuging, and shipping FF for detection have no underlying impact. It is suggested that the above process may still impact oxidized lipid metabolites in FF. Furthermore, because the limited amount of oxidized lipid metabolites that can be detected by oxidized lipid metabolomics at present, we speculate that the different oxidative lipid metabolites in FF of DOR and NOR patients are way much limited, to an extent that the up-regulated metabolites may not be detected. Finally, it is possible that we analyzed a small number of samples and the individual differences of clinical samples were large. Although patients were screened with strict adherence to the inclusion criteria, the influence of individual differences cannot be ignored. Therefore, the difference between DOR and NOR patients in the up-regulated oxidized lipid metabolites in FF is not significant. Another finding of this study is that the down-regulated oxidized lipid metabolites in FF of DOR patients are primarily linked to AA metabolism. Mounting evidence indicates that AA potentially induces and inhibits oxidative stress, for instance, it can increase calcium influx by activating the calcium channel on the cell membrane [59, 60], which subsequently activates NADPH oxidase, increasing ROS levels and induces oxidative stress damage [61]. AA can also activate the mRNA and protein levels of PPAR, ameliorate the activities of SOD, CAT, and GSH-Px, decrease the production of mitochondrial ROS to arrest oxidative stress injury [62]. These data strongly suggest a dominant role for AA and its metabolites in anti-oxidative stress in FF of DOR patients, which will be explored in our future study.

## Conclusions

Through UHPLC-MS/MS, oxylipins metabolites changes in FF of the DOR and NOR patients were investigated. Metabonomic analysis of FF showed 15 differentially expressed oxylipins metabolites, associated with ovarian reserve function. The differentially oxidized lipid metabolites were lower in FF of DOR patients as compared to those of the NOR group. The pathway enrichment analysis demonstrated that the differentially oxidized lipid metabolites are mainly concentrated in the AA metabolic pathway, while AA is involved in the regulation of oocyte development and maturation, and its complex changes are closely related to follicular development. The differential oxylipins metabolites and AA metabolic pathway may play a significant role in DOR infertility. Dysfunctions in the metabolism of oxylipins related to follicular development in FF of DOR patients may provide potential detection avenues and therapeutic targets for promoting oocyte maturation. The findings may also provide a scientific basis for understanding the microenvironment of oocyte development and improving oocyte quality in patients with DOR.

## Data Availability

The datasets used and analyzed during the current study are available from the corresponding author on reasonable request.

## Abbreviations

DOR: Diminished ovarian reserve
POF: Premature ovarian failure
POR: Poor ovarian responder
NOR: Normal ovarian reserve
IVF: In vitro fertilization
ICSI-ET: intracytoplasmic sperm injection-embryo transfer
OS: Oxidative stress
ROS: Reactive oxygen species
MDA: Malondialdehyde
FF: Follicular fluid
AGE: Advanced glycation end-products
AMH: Anti-Mullerian hormone
FSH: Follicle stimulating hormone
LH: Luteinizing hormone
bAFC: Basic antral follicle count
E_2_: Estrogen
P: Progesterone
SWATH: Sequential window acquisition of all theoretical fragment-ion spectra
MRM: Multiple reaction monitor
PCA: Principal component analysis
OPLS-DA: Orthogonal projections to latent structure discriminant analysis
QC: Quality control
VIP: Variable importance in the projection
KEGG: Kyoto Encyclopedia of Genes and Genomes
BMI: Body mass index
HMG: Human menopausal gonadotropin
HCG: Human chorionic gonadotropin
SOD: Superoxide dismutase
CAT: Catalase
GSH-PX: Glutathione peroxidase
AA: Arachidonic acid
FDR: False discovery rate
COX: Cyclooxygenase
LOX: Lipoxygenase
CYP: Cytochrome P450
PUFAs: Polyunsaturated fatty acids
PLA2: Phospholipase A2
CGCs: Cumulus granulosa cells
ASA: Acetylsalicylic acid
20-HETE: 20-hydroxyeicosatetraenoic acid
IR: Insulin resistance
PGs: Prostaglandins
PTGS1: Prostaglandin-endoperoxide synthase S1
PTGS2: Prostaglandin-endoperoxide synthase S2
PPARγ: Peroxisome proliferator-activated receptor γ
ROC: Receiver operating characteristic
AUC: Area under the curve
